# Unusual Presentation of Pustular Psoriasis on the Face: A Case Report

**DOI:** 10.7759/cureus.105317

**Published:** 2026-03-16

**Authors:** Natalia C Guerra, Trevor Nessel, Ali Moiin

**Affiliations:** 1 Internal Medicine, HCA Lawnwood Hospital, Fort Pierce, USA; 2 Dermatology, Beaumont Hospital, Farmington Hills, USA; 3 Dermatology, Wayne State University School of Medicine, Detroit, USA

**Keywords:** acute generalized pustular psoriasis, biologic therapies, generalized pustular psoriasis (gpp), management of pustular psoriasis, palmoplantar pustular psoriasis, spesolimab

## Abstract

This case report describes an unusual presentation of pustular psoriasis on the face in a 36-year-old woman with a history of systemic lupus erythematosus (SLE). The patient presented with a rapidly worsening facial rash initially diagnosed as pustular psoriasis with superimposed bacterial impetiginization. Following ineffective initial treatment, she was started on spesolimab, a newly Food and Drug Administration (FDA)-approved monoclonal antibody targeting IL-36R, which led to rapid clinical improvement. The report highlights the clinical challenges of diagnosing pustular psoriasis, particularly in atypical cases, and underscores the importance of early intervention with targeted therapies to prevent disease progression.

## Introduction

Pustular psoriasis is a rare and severe variant of psoriasis characterized by the presence of sterile pustules on an erythematous base. Unlike the more common plaque psoriasis, pustular psoriasis can present in two distinct forms: localized, typically affecting the palms and soles, and generalized, which can involve widespread areas of the body. The disease is known for its relapsing and remitting course, with potential triggers including infections, medications, and other underlying health conditions [1-3]. Despite its well-documented clinical patterns, pustular psoriasis involving the face is exceedingly rare, posing a diagnostic challenge for clinicians.

This case report details the presentation, diagnosis, and management of a 36-year-old woman with a history of systemic lupus erythematosus (SLE) who developed an unusual facial rash that was ultimately diagnosed as pustular psoriasis. The patient's case is notable not only for the rare facial involvement but also for the therapeutic response to spesolimab, a newly approved monoclonal antibody specifically targeting the IL-36 receptor [4]. This introduction aims to provide a context for understanding the clinical significance of this case, emphasizing the importance of recognizing atypical presentations of pustular psoriasis and the evolving treatment landscape for this challenging dermatological condition.

## Case presentation

**Figure 1:**
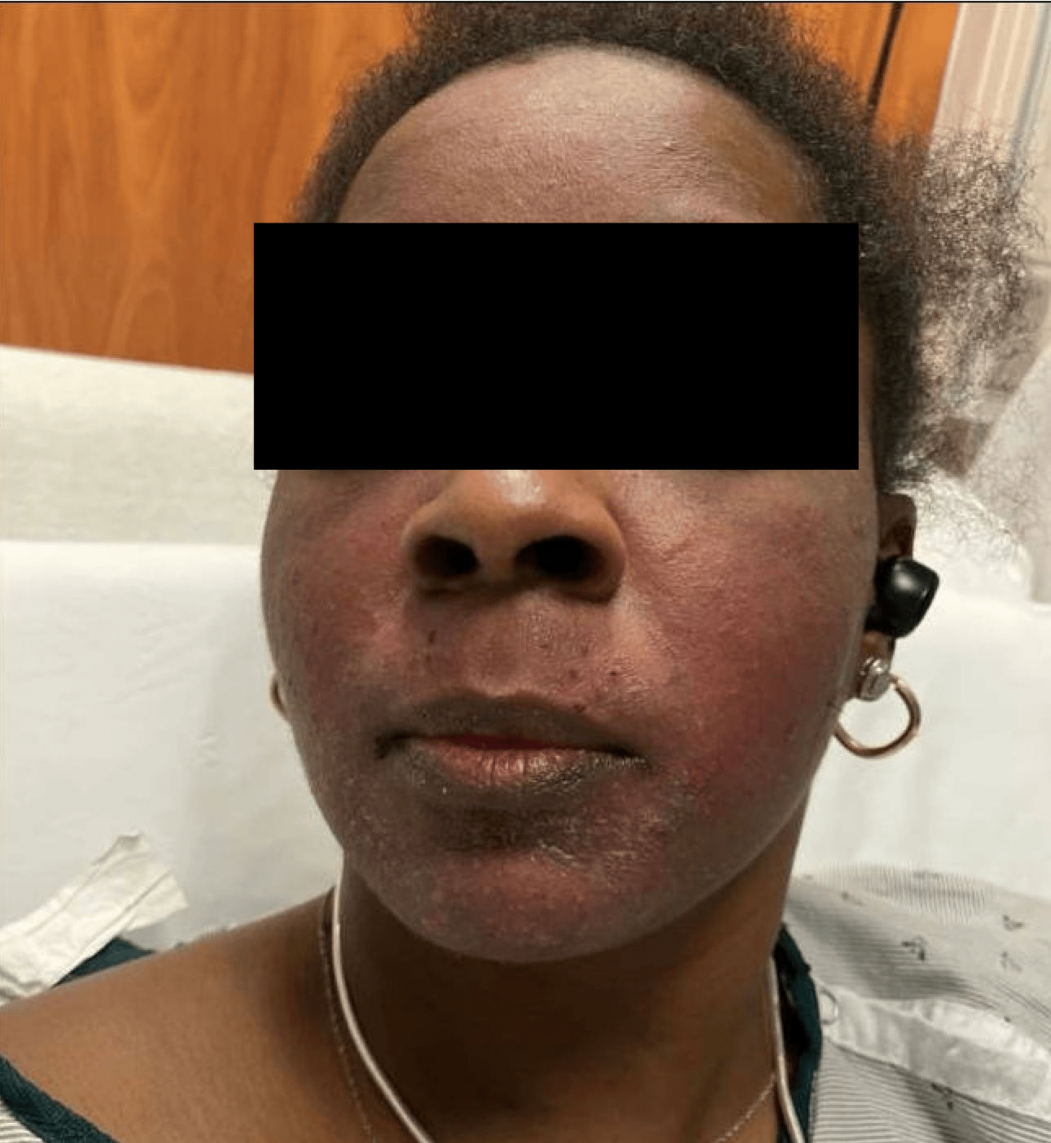
Facial rash beginning in a perioral and submental distribution

**Figure 2:**
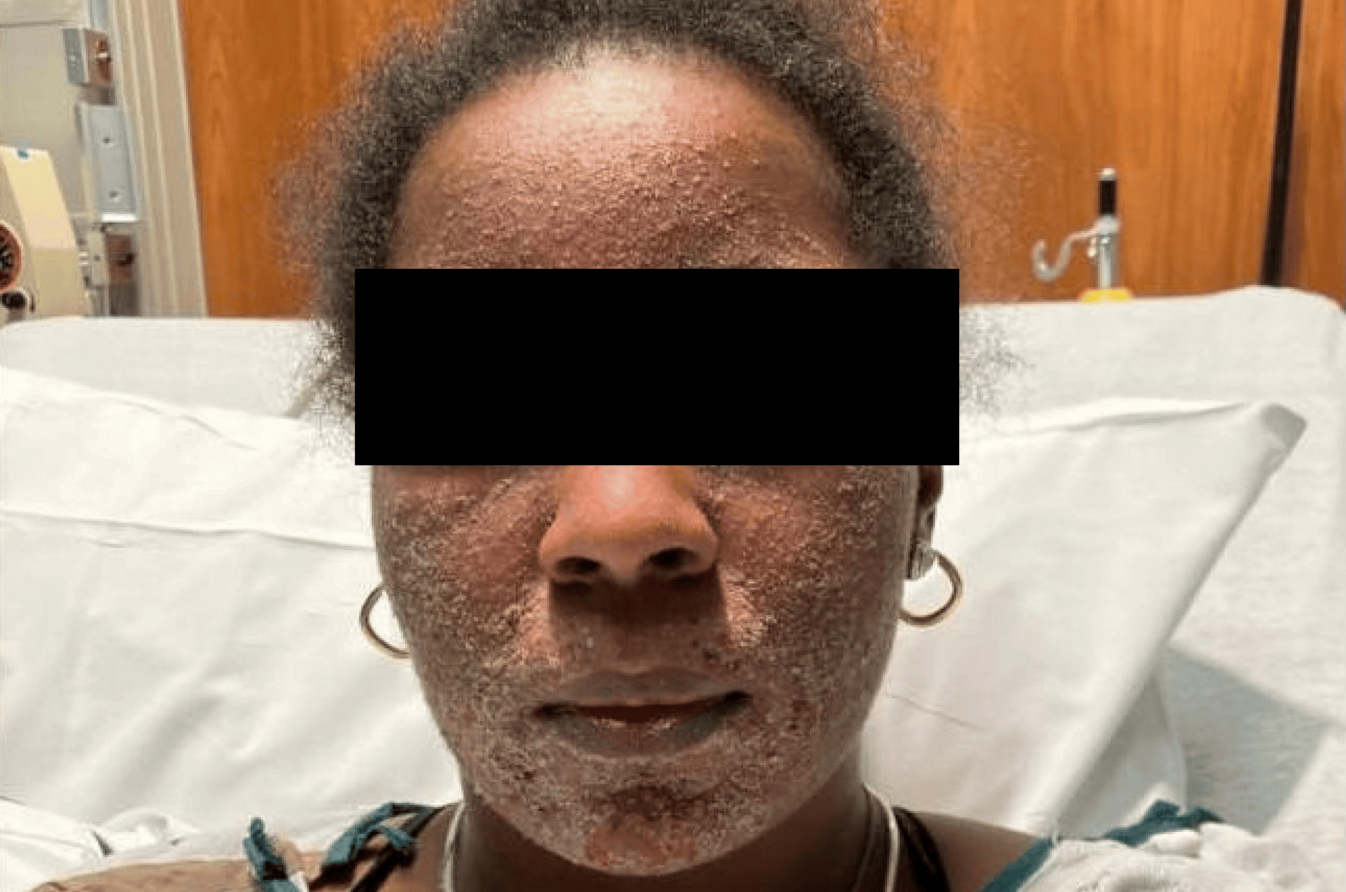
Facial rash upon progression

A 36-year-old woman with a medical history of SLE on hydroxychloroquine 200 mg twice daily presented to the emergency department (ED) for a facial rash. The rash began as a few pustules under her chin (Figure 1) and progressively worsened to cover the entire face. She was seen by her dermatologist six days after the rash initially presented, at which time a skin biopsy was performed along with a herpes simplex virus (HSV)/varicella-zoster virus (VZV) polymerase chain reaction (PCR) swab and a bacterial wound culture. The patient was prescribed a five-day course of cephalexin, mupirocin, and prednisone 20 mg three times daily and instructed to report to the ED if her condition worsened, which prompted her current presentation. Dermatology was consulted due to concern for pustular psoriasis. The patient was afebrile, and other vitals were within normal limits. On examination, there were crusted papules and pustules coalescing into plaques on the face (Figure 2).

Results from the outpatient biopsy one week prior were reviewed in the ED, revealing marked neutrophilic dermal and epidermal inflammation with pustular formation. The adjacent epidermis showed spongiosis, mild hyperkeratosis, and minimal hyperplasia, consistent with pustular psoriasis. HSV 1 and 2 and VZV PCR were negative. Immunohistochemistry and immunofluorescence were negative for A1-2, A1-3, A1-4, A1-5, A1-6, and A1-8. Periodic acid-Schiff (PAS) staining was negative. Results for bacterial culture were still pending at the time. The patient was diagnosed with pustular psoriasis with suspected superimposed bacterial impetiginization. She was started on doxycycline 100 mg twice daily for 10 days and topical mupirocin three times daily and advised to continue prednisone 16-day taper.

About two weeks later, the rash had spread to the entire body. Bacterial cultures were reviewed and were negative. The patient was subsequently started on spesolimab infusions with rapid clinical improvement. At both six-month and one-year follow-ups, she was without any clinical recurrence. 

## Discussion

Pustular psoriasis is a form of psoriasis that often poses clinical challenges to dermatologists and other healthcare providers. It classically presents as pustules overlying an erythematous base and has two distinct subtypes: localized and generalized. The localized variant presents on the hands and feet and is referred to as palmoplantar pustulosis (PPP), whereas generalized pustular psoriasis (GPP) is more widespread. Involvement of the fingers, toes, and nails is considered to be a subset of PPP and is considered a separate entity called acrodermatitis continua of Hallopeau (ACH). The disease usually follows a relapsing and remitting course, and triggers such as smoking, infection, medication withdrawal (systemic corticosteroids, cyclosporine, methotrexate), and pregnancy have been identified [1-3].

Pustular psoriasis is different from the more common plaque psoriasis not only clinically but also histologically and genetically [1]. Additionally, there is a difference in the pathogenesis of de novo pustular psoriasis and pustular psoriasis presenting in the setting of a pre-existing plaque psoriasis. Mutations in the IL-36RN gene play a significant role in the development of pustular psoriasis [2,3], with both familial and sporadic loss-of-function mutations reported. Over 50% of de novo GPP cases have homozygous or compound heterozygous IL-36RN mutations, and recessive mutations can cause GPP over pre-existing psoriasis vulgaris (PV). There are 25 reported pathogenic IL-36RN variants. A study found that all GPP-only patients had pathogenic IL-36RN variants, whereas only a few GPP and PV patients had similar mutations. A meta-analysis revealed that IL-36RN mutations are linked to earlier disease onset and lower prevalence of PV [2]. CARD14 mutations appear to predispose those with plaque psoriasis to pustular psoriasis and are rarely seen in patients with de novo GPP [2,3]. It's known that plaque psoriasis is driven by inflammatory pathways involving TNF-α/IL-17/IL-22/IL-23, whereas pustular psoriasis is driven by the unopposed activation of IL-36R due to loss-of-function mutation of IL-36a. Gene profiling of skin biopsies from patients with pustular psoriasis showed higher expression of IL-36α, IL-36γ, and IL-1β and lower expression of IFNγ and IL-17A than those with plaque psoriasis. Pustular psoriasis biopsies have also shown increased expression of chemokines directed at neutrophils compared to biopsied plaque psoriasis lesions [2]. 

Acute GPP typically presents with sudden-onset burning and erythema of the skin followed by pustular eruption. The erythema and pustulation progressively spread, and the pustules begin to coalesce into lakes of pus that begin to exfoliate, leading to the presence of lesions in different stages. The tongue is notably involved. PPP commonly presents with painful erythema at the tip of the digit following the development of painful pustules around and below the nail bed and matrix. Localized pustular psoriasis (LPP) tends to progress proximally over time, involving parts of the hand or foot [2]. Neither GPP nor PPP traditionally presents on or involves the face, making our patient's presentation unusual. Pustular psoriasis has a mortality rate up to 5% [2], making proper diagnosis and management of utmost importance. 

Due to the differing underlying pathogenesis behind the inflammatory pathways of GPP and PV, traditional immunosuppressants and biologics used to treat PV are not as effective in the treatment of GPP. The abnormal signaling of the IL-36 pathway in GPP makes it a key therapeutic target for the condition. Spesolimab, originally approved by the Food and Drug Administration (FDA) for the treatment of GPP flares in adults in 2022, is a first-in-class humanized monoclonal antibody that binds specifically to the IL-36 receptor, therefore directly antagonizing IL-36 signaling. Recently, spesolimab was approved for subcutaneous dosing for GPP in patients not experiencing a flare [4].

Clinical evidence supporting the use of spesolimab has been demonstrated in proof-of-concept studies and in the randomized, placebo-controlled EFFISAYIL-1 trial. In this phase 2 study, patients presenting with an acute GPP flare were randomized to receive a single intravenous dose of spesolimab or placebo, with the option to receive an additional dose if symptoms persisted. The primary endpoint of the study was a Generalized Pustular Psoriasis Physician Global Assessment (GPPGA) pustulation subscore of 0, indicating complete pustular clearance, at week 1. A significantly greater proportion of patients treated with spesolimab achieved this endpoint compared with those receiving placebo. Treatment with spesolimab was also associated with rapid improvement in overall disease severity as measured by the total GPPGA score. In addition to improvements in cutaneous findings, patients treated with spesolimab also demonstrated improvement in systemic manifestations associated with GPP flares, including fever and markers of systemic inflammation, and the treatment demonstrated an acceptable safety profile in the study population [5].

## Conclusions

It is imperative for dermatologists and other healthcare providers to include pustular psoriasis in their differential diagnosis when evaluating a patient with or without a history of plaque psoriasis presenting with a pustular rash localized to the face. This case emphasizes the necessity of maintaining a broad differential diagnosis, even in atypical presentations, and highlights the importance of performing a skin biopsy to accurately diagnose atypically presenting pustular psoriasis. Additionally, this case suggests that it may be beneficial to start treatment with spesolimab early in the disease process to prevent the spread of GPP from one body region to another, as was seen in our patient. 
